# The Influence of Genetics in the Early Development of Axial Postural Abnormalities in Parkinson's Disease

**DOI:** 10.1002/mdc3.70290

**Published:** 2025-08-13

**Authors:** Ilaria A. Di Vico, Silvia Gallo, Eleonora Bertoncello, Claudia Ledda, Michele Tinazzi, Leonardo Lopiano, Carlo Alberto Artusi

**Affiliations:** ^1^ Neurology Unit, Borgo Roma Hospital; Department of Neurosciences, Biomedicine and Movement Sciences Policlinico Borgo Roma, University of Verona Verona Italy; ^2^ Department of Neuroscience “Rita Levi Montalcini” University of Turin SC Neurologia 2U, AOU Città della Salute e della Scienza Turin Italy

**Keywords:** Parkinson's disease, genetics, postural abnormalities, GBA, SNCA

## Abstract

**Background:**

Postural abnormalities (PA) can complicate Parkinson's disease (PD). While age and motor severity are established predictors, the genetic role remains underexplored.

**Objective:**

To evaluate the influence of major genetic variants on PA development in PD over 4 years.

**Methods:**

We analyzed 429 patients from Parkinson's Progression Markers Initiative, including *GBA*, *LRRK2* and *SNCA* mutation carriers. PA were assessed using the MDS‐UPDRS‐III item 3.13 and risk factors were analyzed with Cox uncertain regression.

**Results:**

SNCA‐PD patients were the youngest at onset (50.8 years) and showed the highest PA cumulative incidence over 4 years (30%), followed by GBA‐PD (25.8%), idiopathic PD (23%), and LRRK2‐PD (17.2%).

No significant differences in PA prevalence were found across groups at baseline or at the 4‐year follow‐up, and genetic status was not a predictor for PA development.

**Conclusions:**

Although not significant, the higher PA incidence in SNCA‐PD, despite its younger age, suggests that genetic factors may influence PA progression, warranting further studies.

Axial postural abnormalities (PA), namely camptocormia, antecollis and Pisa syndrome, represent a relevant motor complication in patients with Parkinson's Disease (PD).^1^ They usually occur in advanced disease stages and are linked to increased falls and loss of autonomy,^2^ leading to reduced quality of life.^3^ Recent studies have focused on PA epidemiology, pathophysiology,^4^ and risk factors,^5^ aiming to identify characteristics that predispose individuals to develop these complications. Central and peripheral mechanisms, respectively dystonia, proprioception, visuospatial and vestibular impairment and myopathy contribute to PA occurrence.^4^ Older age at baseline and higher motor symptoms severity have been found to be the strongest predictors of PA development.^6^ Despite advances in understanding PA predictors, the role of genetic mutations in influencing their development remains underexplored.

*GBA* variants, the strongest genetic risk factors for PD, are typically associated with a more severe disease course,^7^ but evidence directly linking these mutations to PA is currently limited. Biallelic *PRKN* variants, the most common genetic cause of early‐onset slowly progressing parkinsonism, have been linked to postural deformities development, particularly in later stages. *SNCA* mutations, with a “dosage effect” depending on gene copies number, are known to contribute to severe motor phenotypes,^8^ sometimes including axial deformities. Finally, while *LRRK2*‐PD often resembles sporadic PD, pathogenic variants in this gene seem to increase the risk of postural instability and gait difficulties (PIGD), which may predispose patients to PA.^9^, ^10^

This study leverages data from Parkinson's Progression Markers Initiative (PPMI),^11^ a multicenter longitudinal study collecting clinical information from a large PD cohort with prospective data collection. We aimed to evaluate the relationship between specific genetic variants and risk of developing PA.

## Methods

### Study Population

PPMI is a multicentric, longitudinal cohort study enrolling patients with no more than two‐year PD diagnosis, confirmed by clinical examination and DaT‐SPECT imaging. Our cohort consists of PD patients from the PPMI dataset who underwent genetic analysis for *GBA*, *LRRK2*, *PRKN*, and *SNCA* variants. Patients were assessed at baseline and followed‐up annually with clinical and demographic data collection. In this study we included only PPMI participants with complete data at baseline and 4‐year follow‐up from their initial assessment for whom genetic analysis and motor assessment (MDS‐UPDRS‐III) were available.

### Genetic Subtyping

Participants were categorized into four groups based on genetic status: patients with pathogenic variants in *GBA*, *LRRK2*, and *SNCA* and patients without identified variants (idiopathic‐PD). Genetic variant pathogenicity was determined using established databases and literature references.^7^, ^12^ Patients with heterozygous *PRKN* variants (*n* = 10) were excluded due to the unclarified role in PD pathogenesis.^13^, ^14^

### Clinical and Posture Evaluation

Posture was assessed using MDS‐UPDRS‐III item 3.13 in OFF‐medication state in order to minimize treatment confounding effects. Presence of axial PA was defined as a cut‐off score ≥2 (“Definite flexion, scoliosis or leaning to one side, but patient can correct posture to normal posture when asked to do so”).

The clinical phenotype, reflecting severity and presentation of motor and non‐motor symptoms, was classified into malignant, benign, or intermediate using a validated scoring system.^15^ Other relevant demographic and clinical features collected at baseline and after a 4‐years follow‐up were: sex, age, MDS‐UPDRS‐III total score, Hoehn and Yahr (H&Y), and Levodopa Equivalent Daily Doses (LEDD)—divided into total LEDD, levodopa‐LEDD, and dopamine‐agonist‐LEDD (DA‐LEDD).^16^

### Statistical Analysis

We used descriptive statistics for continuous variables and frequency for categorical ones. Comparison between groups in main demographic and clinical variables was performed with Kruskal–Wallis test for continuous variables, Tukey test for ordinal ones, and Fisher's exact test for dichotomic ones. For the entire population and then for each genetic subgroup, we calculated PA cumulative incidence, considered as number of new PA over the four‐years follow‐up, incidence rates, calculated based on the total number of person‐years at risk, and PA prevalence, considered as proportion of patients with PA on the total number of patients, both at baseline (within 1–2 years from disease onset) and at last follow‐up (4–6 years of disease). Comparison between groups at baseline and after 4 years in PA prevalence was performed with Fisher's exact test, while the log‐rank test was used to assess differences in cumulative PA incidence. Factors influencing the risk of developing PA over 4 years were explored with Cox proportional hazards regression. PA occurring after baseline was the dependent variable, while key independent variables included genetic variant status (categorical: GBA, LRRK2, SNCA, no variant), and two variables already known as predictors of PA development^6^: age at baseline and baseline MDS‐UPDRS‐III. *p* values are two‐tailed, with a cut‐off level of significance 0.05. Analyses were performed using SPSS software (IBM 27).

## Results

### Demographic and Clinical Characteristics

According to inclusion/exclusion criteria, we included in the analysis 429 PD patients, divided into four groups: 37 patients with *GBA* variants, 71 with *LRRK2* variants and 12 with *SNCA* variants (Table 1). No patients with homozygous PRKN variants were found. Within *GBA* group, 81.1% (*n* = 30) carried the mild N409S variant, whereas 16.2% (*n* = 6) carried severe variants, namely R159W and L483P (Table 1).

**TABLE 1:** Demographics, clinical, genetic features of the PD cohort, incidence, and prevalence of postural abnormalities

Baseline measures	All patients (*n* = 429)	I‐PD (*n* = 309)	GBA (*n* = 37)	LRRK2 (*n* = 71)	SNCA (*n* = 12)	*p*‐value (overall)	*p* value (pairwise)
Sex (M/F)	269/160	208/101	22/15	35/36	4/8	**0.005**	**ID ‐ LRRK2 (*p* = 0.004); ID** ‐ **SNCA (*p* = 0.015)**.
Age (years)	61.1 ± 9.9	61.4 ± 9.8	60.2 ± 11.1	62.1 ± 8.6	50.8 ± 11.6	**0.013**	**SNCA<ID (*p* = 0.002); SNCA<GBA (*p* = 0.018); SNCA<LRRK2 (*p* = 0.001)**.
MDS‐UPDRS‐III	20.7 ± 9.5	20.3 ± 8.7	23.8 ± 11.2	20.9 ± 10.3	20.7 ± 16.3	0.294	**No significant differences**
Item 3.13	0.64 ± 0.68	0.64 ± 0.67	0.84 ± 0.69	0.54 ± 0.71	0.67 ± 0.78	0.182	**No significant differences**
Phenotype	0.79 ± 0.72	0.71 ± 0.68	1.03 ± 0.76	0.97 ± 0.8	1.09 ± 0.8	**0.003**	**ID<LRRK2 (*p* = 0.033)**.
H&Y	1.6 ± 0.53	1.53 ± 0.5	1.64 ± 0.64	1.86 ± 0.51	1.75 ± 0.75	**<0.001**	**ID<LRRK2 (*p <* 0.001)**.
LEDD L‐Dopa (mg)	151.8 ± 313.2	91.4 ± 239	265.1 ± 441.7	333.6 ± 393.4	284 ± 466.2	**<0.001**	**ID<GBA (*p* = 0.002); ID<LRRK2 (*p* < 0.001); GBA<LRRK2 (*p* = 0.022)**.
LEDD DA (mg)	50 ± 130.6	35.1 ± 127.2	64.6 ± 125.4	86.6 ± 133.6	174 ± 117.6	**<0.001**	**ID<GBA (*p* = 0.034); ID<LRRK2 (*p* < 0.001); ID<SNCA (*p* < 0.001); LRRK2 < SNCA (*p* < 0.001); GBA<SNCA (*p* < 0.001)**.
LEDD (mg)	201.9 ± 347.9	126.5 ± 268.8	329.8± 466.9	420.1 ± 417.1	458 ± 521	**<0.001**	**ID<GBA (*p* = 0.001); ID<LRRK2 (*p* < 0.001); ID<SNCA (*p* < 0.001); GBA<LRRK2 (*p* = 0.012); GBA<SNCA (*p* = 0.016)**.
Genetic variants	‐	‐	N409S:*30 (81%)* L483P: *5 (13%)* R159W: *1 (3%)* IVS2 + 1G>A: *1 (3%)*	G2019S: *59 (83%)* R1441G: *12 (17%)*	A53T: *12 pts (100%)*	‐	‐
Cumulative incidence (%)	22.5%	23%	25.8%	17.2%	30%	0.328	‐
Incidence rates (per 100 PY)	5.6	5.8	6.5	4.3	7.5	‐	‐
Prevalence PA baseline (%)	48 (11.2%)	33 (10.7%)	6 (16.2%)	7 (9.9%)	2 (16.7%)	0.68	**No significant differences**
Prevalence PA 4 years (%)	137 (32%)	99 (32%)	15 (40.5%)	18 (25.3%)	5 (41.7%)	0.362	**No significant differences**

*Note*: The mean ± standard deviation is reported for each variable. Bold *p*‐values indicate statistical significance <0.05. Post‐hoc analyses specify pairwise differences. Cumulative incidence is expressed as the percentage of new PA cases among patients at risk over the 4‐year follow‐up period. The incidence rate is expressed as the number of new PA cases per 100 person‐years of observation. *P*‐values were calculated using Fisher's exact test for prevalence and the log‐rank test for cumulative incidence.

Abbreviations: DA: dopamine agonist; GBA: Glucocerebrosidase; H&Y: Hohen and Yahr; i‐PD: Idiopathic PD patients; LEDD: levodopa equivalent daily dose; LRRK2: Leucine‐Rich Repeat Kinase 2; MDS‐UPDRS: Movement Disorders Society‐Unified Parkinson's Disease Rating Scale; PA: postural abnormalities; PY: person‐years; SNCA: Alpha‐Synuclein.

Mean age at diagnosis differed significantly across groups (*p* = 0.013), with *SNCA*‐PD showing the youngest age at onset (50.8 years) compared to idiopathic PD (i‐PD, 61.4 years), *GBA*‐PD (60.1 years) and *LRRK2*‐PD (62.1 years) (Table 1). No statistically significant differences were observed in motor symptom severity between groups, as assessed by MDS‐UPDRS‐III total score (*p* = 0.294). PD phenotype had significant differences across groups (*p* = 0.003), with *LRRK2* showing a higher proportion of malignant phenotype when compared to i‐PD (Table 1). Furthermore, H&Y staging revealed significantly more advanced disease in *LRRK2* and *SNCA* variant carriers compared to i‐PD patients (𝑝 < 0.001). Moreover, genetically mutated patients required significantly higher mean therapeutic doses than i‐PD ones, in particular those with SNCA and LRRK2 variants (Table 1).

### Axial Postural Abnormalities in Genetic Subgroups

PA prevalence was 11.2% for the entire cohort at baseline, without statistically significant differences across groups (𝑝 = 0.680), and 32% at 4 years, again without significant differences (𝑝 = 0.362).

The 4‐year cumulative incidence of PA was 22.5% for the entire cohort, 23% for i‐PD group, 25.8% for *GBA*, 17.2% for *LRRK2*, and 30% for *SNCA* (Table 1). Log‐rank test showed no significant differences in cumulative incidence among groups (𝑝 = 0.328).

Cox regression analysis, adjusted for age and baseline MDS‐UPDRS‐III scores, did not disclose genetic status as a significant determinant for PA development (𝑝 = 0.742), while age and baseline MDS‐UPDRS‐III scores were confirmed as significant predictors of postural abnormality progression (Table 2).

**TABLE 2:** Hazard ratios for postural abnormalities by genetic group, age, and baseline disease severity

Variables	*p* value	HR	95% C.I.
Idiopathic PD	0.176		
GBA	0.616	1.229	0.55–2.746
LRRK2	0.191	0.658	0.351–1.233
SNCA	0.109	3.46	0.758–15.804
Age	<0.001	1.07	1.042–1.098
UPDRS‐III basal	<0.001	1.077	1.05–1.104

*Note*: This table presents the results of the Cox regression analysis, including hazard ratios (HR), and 95% confidence intervals (C.I.) for genetic variants (GBA, LRRK2, SNCA), age, and baseline UPDRS III scores, and p‐values. Idiopathic PD patients are used as the reference group. The hazard ratio (HR) indicates the relative risk of postural abnormalities compared to the reference group. Alpha‐Synuclein (SNCA), Glucocerebrosidase (GBA), Leucine‐Rich Repeat Kinase 2 (LRRK2), Idiopathic Parkinson's Disease (ID).

## Discussion

The primary objective of this study was to evaluate the relationship between the most frequent PD‐related genes, *GBA*, *LRRK2*, and *SNCA*, and the prevalence of axial PA, whereas the secondary one was to compare incidence and prevalence of PA between different genetic subgroups over a 4‐year follow‐up period. The overall prevalence of PA across all PD patients was 11.2% at baseline and 32% at the 4‐year follow‐up, without statistically significant differences between the genetic subgroups both at baseline (*p* = 0.680) and at 4 years (*p* = 0.362).

At baseline, PA prevalence was the highest in *SNCA*‐PD (16.7%), followed by *GBA* (16.2%), i‐PD (10.7%), and *LRRK2* (9.9%). At the 4‐year follow‐up, PA prevalence increased in all subgroups, with the SNCA group again showing the highest prevalence (41.7%), followed by *GBA* (40.5%), i‐PD (32%), and *LRRK2* (25.3%). Although these differences are not statistically significant, they suggest that some genetic variants, particularly in *SNCA*, may be associated with a slightly higher likelihood of presenting PA at an early PD stage. Specifically, SNCA‐PD showed a trend of higher incidence of PA, with a relative risk approximately three times greater than GBA‐PD (based on hazard ratio values), indicating that SNCA may play a role in early PA progression. Further studies are needed to confirm this potential trend.

Notably, SNCA‐PD patients were the youngest at disease onset, and the higher PA prevalence in this group may reflect a genetic contribution to PA development that is, at least partially, independent of age. However, the small sample size of the *SNCA* group (*n* = 12), as well as the limited number of patients in the *GBA* and *LRRK2* subgroups, may have reduced the statistical power of the analysis and contributed to the lack of significant group differences, thus limiting the generalizability of these findings. Although genetic status did not appear to be a significant determinant for PA development, a higher cumulative incidence of PA during the 4‐year follow‐up was observed in *SNCA* and *GBA* groups (30% and 25.8%, respectively), compared to 23% in i‐PD and 17.2% in *LRRK2*‐PD. This result is intriguing, given that, according to previous studies, genetic mutations can influence various aspects of disease progression, including motor symptoms and motor complications. It is noteworthy that *LRRK2* variant carriers have a low incidence of PA over time, despite showing a similar motor symptom severity than other groups and a higher proportion of malignant phenotype than idiopathic PD. Interestingly, LRRK2 variant carriers exhibited a lower incidence of PA over time, despite showing similar motor symptom severity to other groups. This may suggest a potential protective role of LRRK2 mutations in modulating the development of axial postural abnormalities in PD or a different phenotype expressed by LRRK2 carriers,^17^ not including early onset of PA, although further studies are required to substantiate this hypothesis. Lack of statistically significant differences indicates that, in this cohort of patients who are predominantly in the early PD stages, age and baseline motor severity (indicated by MDS‐UPDRS‐III) may be more relevant predictors of PA than genetic factors. The multifactorial nature of PA, consisting of central (ie, neurodegeneration and severity of motor symptoms) and peripheral (ie, degenerative musculoskeletal alterations) pathogenic mechanisms, could further explain our results,^4^ since these factors accumulate with age and disease progression increasing the likelihood of developing PA.^6^

Moreover, in our cohort, *LRRK2* mutations exhibited a lower tendency to develop PA during the 4‐year follow‐up, suggesting that PA in *LRRK2*‐PD take a longer time to manifest compared to other genetic forms or i‐PD. This aligns with previous studies indicating that *LRRK2*‐PD progresses more slowly with a more gradual onset of motor symptoms, like postural instability and gait difficulties, especially in people with late‐onset disease.

This study also found a trend towards a higher incidence of PA in *GBA* mutation carriers compared to i‐PD and *LRRK2* carriers. *GBA* mutations are linked to earlier and more severe motor symptoms, such as bradykinesia and rigidity, which may contribute to PA development. These findings suggest that *GBA* mutations in PD may play a significant role in accelerating motor complications, including PA.

While the results of this study provide valuable, yet preliminary, insights, several aspects warrant further investigation. As PA typically manifest in more advanced stages, our cohort might not fully represent the later disease stages where these abnormalities become more pronounced, especially considering the early stage of disease at enrollment and the limited 4‐year follow‐up. Nevertheless, the relatively high rate of PA in all patients, including those with genetic mutations (such as *GBA* and *SNCA*) and i‐PD, suggests that PA development may occur earlier and more frequently than traditionally assumed. One of the main limitations of our study is the restricted focus to a subset of PD‐related genetic variations, while other potentially relevant mutations such as *PRKN*, *VPS35*, and others were not included. This limited genetic stratification may have led to an underestimation of genotype‐specific effects on PA development. Future studies including larger sample size and a broader range of genes may provide a more comprehensive understanding of how genetic factors influence the development of PA and other motor complications in PD. Additionally, our study did not include data on musculoskeletal alterations, non‐motor symptoms, or concomitant medications, which may act as confounding factors in the development of postural abnormalities. Finally, the assessment of PA relied solely on MDS‐UPDRS item 3.13, without additional biomechanical or radiological validation.

In conclusion, limitations notwithstanding, our findings suggest that *GBA*, *LRRK2*, and *SNCA* mutations do not significantly predict axial PA development in the early phase of PD, but trends suggest that *GBA* and *SNCA* may be linked to a higher incidence of PA, showing that genetic factors, along with age and baseline motor severity, could influence PA progression. The lack of statistically significant differences highlights the need for larger studies with longer follow‐up to better understand the role of genetics in PA development. Further analysis of other genetic variants could offer additional insights.

## Author Roles

Research Project: A. Conception, B. Organization, C. Execution. Statistical Analysis: A. Design, B. Execution, C. Review and Critique. Manuscript Preparation: A. Writing of the first draft, B. Review and Critique.

I.A.D.V.: 1B, 1C; 2A; 3A, 3B.

S.G.: 1B, 1C; 2A; 3A, 3B.

E.B.: 1B, 1C; 2A; 3A, 3B.

C.L.: 1B; 2B, 2C; 3B.

L.L.: 1A, 1B; 2A, 2C; 3B.

M.T.: 1A, 1B; 2C; 3B.

C.A.A.: 1A, 1B, 1C; 2A, 2B, 2C; 3B.

## Disclosures

**Ethical Compliance Statement:** Ethical standards This study is based on data from “The Parkinson's Progression Markers Initiative (PPMI),” which is a longitudinal study performed in accordance with the ethical standards laid down in the 1964 Declaration of Helsinki and its later amendments and has obtained ethic committee approval from all enrolling centers. Before entering the study, all participants gave their written informed consent. The authors of the current study are not part of the PPMI initiative, and asked and obtained access to anonymized data, as per the rules established and approved of the PPMI initiative. We confirm that we have read the Journal's position on issues involved in ethical publication and affirm that this work complies with those guidelines.

**Funding Sources and Conflicts of Interest:** No specific funding was received for this work. The authors declare that there are no conflicts of interest relevant to this work.

**Financial Disclosures (Last 12 months):** CAA, MT and LL received speaker honoraria and travel grants from Bial, Abbvie, Zambon, Ralpharma. No additional disclosures to report.

## Data Availability

The data that support the findings of this study are available on request from the corresponding author. The data are not publicly available due to privacy or ethical restrictions.

## References

[1] Doherty KM , van de Warrenburg BP , Peralta MC , Silveira‐Moriyama L , Azulay JP , Gershanik OS , Bloem BR . Postural deformities in Parkinson's disease. Lancet Neurol 2011;10:538–549. 21514890 10.1016/S1474-4422(11)70067-9

[2] Geroin C , Artusi CA , Gandolfi M , et al. Does the degree of trunk bending predict patient disability, motor impairment, falls, and back pain in Parkinson's disease? Front Neurol 2020;11:207. 32296383 10.3389/fneur.2020.00207 PMC7136533

[3] Tinazzi M , Gandolfi M , Ceravolo R , et al. Postural abnormalities in Parkinson's disease: an epidemiological and clinical multicenter study. Movement Disorders Clinical Practice 2019;6(7):576–585. 10.1002/mdc3.12810. 31538092 PMC6749805

[4] Artusi CA , Geroin C , Nonnekes J , et al. Predictors and pathophysiology of axial postural abnormalities in parkinsonism: a scoping review. Mov Disord Clin Pract 2023;10(11):1585–1596. 10.1002/mdc3.13879. 38026508 PMC10654876

[5] Geroin C , Artusi CA , Nonnekes J , et al. Axial postural abnormalities in parkinsonism: gaps in predictors, pathophysiology, and management. Mov Disord 2023;38(5):732–739. 37081741 10.1002/mds.29377

[6] Fabbri M , Campisi C , Ledda C , et al. Incidence and predictors of postural abnormalities in Parkinson's disease: a PPMI cohort study. J Neurol 2024;271:4628–4634. 10.1007/s00415-024-12457-3. 38796527

[7] Petrucci S et al. GBA‐related Parkinson disease: dissection of genotype‐phenotype correlates in a large Italian cohort. Mov Disord 2020;35(9):1759–1770. 10.1002/mds.28195. 32658388

[8] Guadagnolo D , Piane M , Torrisi MR , Pizzuti A , Petrucci S . Genotype‐phenotype correlations in monogenic Parkinson disease: a review on clinical and molecular findings. Front Neurol 2021;12:648588. 10.3389/fneur.2021.648588. 34630269 PMC8494251

[9] Palakurthi B , Burugupally SP . Postural instability in Parkinson's disease: a review. Brain Sci 2019;9:239. 10.3390/brainsci9090239. 31540441 PMC6770017

[10] Nabli F , Sassi SB , Amouri R , Duda JE , Farrer MJ , Ben Sassi S , Hentati F . Motor phenotype of LRRK2‐associated Parkinson's disease: a Tunisian longitudinal study. Mov Disord 2015;30(2):253–258. 25487881 10.1002/mds.26097

[11] Data used in the creation of this article were obtained on 10/31/2024 from the Parkinson's Progression Markers Initiative (PPMI) database; (www.ppmi-info.org/access-data-specimens/download-data), RRID:SCR_006431. For up‐to‐date information on the study, visit www.ppmi-info.org.

[12] Richards S , Aziz N , Bale S , et al. Standards and guidelines for the interpretation of sequence variants: a joint consensus recommendation of the American College of Medical Genetics and Genomics and the Association for Molecular Pathology. Genet Med 2015;17(5):405–424. 10.1038/gim.2015.30. 25741868 PMC4544753

[13] Castelo Rueda MP , Raftopoulou A , Gögele M , et al. Frequency of heterozygous Parkin (PRKN) variants and penetrance of Parkinson's disease risk markers in the population‐based CHRIS cohort. Front Neurol 2021;12:706145. 10.3389/fneur.2021.706145. 34434164 PMC8382284

[14] Zhu W , Huang X , Yoon E , et al. Heterozygous PRKN mutations are common but do not increase the risk of Parkinson's disease. Brain 2022;145(6):2077–2091. 10.1093/brain/awab456. 35640906 PMC9423714

[15] Fereshtehnejad SM , Zeighami Y , Dagher A , Postuma RB . Clinical criteria for subtyping Parkinson's disease: biomarkers and longitudinal progression. Brain 2017;140(7):1959–1976. 10.1093/brain/awx118. 28549077

[16] Jost ST , Kaldenbach MA , Antonini A , et al. Levodopa dose equivalency in Parkinson's disease: updated systematic review and proposals. Mov Disord 2023;38(7):1236–1247. 10.1002/mds.29410. 37147135

[17] Saunders‐Pullman R , Mirelman A , Alcalay RN , et al. Progression in the LRRK2‐Asssociated Parkinson disease population. JAMA Neurol 2018;75(3):312–319. 10.1001/jamaneurol.2017.4019. 29309488 PMC5885854

